# Native Chemical Ligation: A Boon to Peptide Chemistry

**DOI:** 10.3390/molecules190914461

**Published:** 2014-09-12

**Authors:** Parashar Thapa, Rui-Yang Zhang, Vinay Menon, Jon-Paul Bingham

**Affiliations:** Department of Molecular Biosciences and Bioengineering, University of Hawaii at Manoa, Honolulu, HI 96822, USA

**Keywords:** native chemical ligation, cyclotides, conotoxin, convergent, sequential, auxiliary, thioester

## Abstract

The use of chemical ligation within the realm of peptide chemistry has opened various opportunities to expand the applications of peptides/proteins in biological sciences. Expansion and refinement of ligation chemistry has made it possible for the entry of peptides into the world of viable oral therapeutic drugs through peptide backbone cyclization. This progression has been a journey of chemical exploration and transition, leading to the dominance of native chemical ligation in the present advances of peptide/protein applications. Here we illustrate and explore the historical and current nature of peptide ligation, providing a clear indication to the possibilities and use of these novel methods to take peptides outside their typically defined boundaries.

## 1. Introduction

Peptides have been at the forefront of scientific research for the past several decades. Their structural diversity and high level of target selectivity position them as prime candidates for therapeutic development. To this end, it is desirable to be able to synthesize peptides under controlled laboratory conditions using highly efficient chemical reactions. The field of synthetic peptide chemistry was first made feasible by the development of Solid Phase Peptide Synthesis (SPPS) in the 1960s by Bruce Merrifield [1], for which he was awarded the 1984 Noble Prize in Chemistry. In addition to making peptide synthesis practical, this technique allows for the introduction of non-native amino acids and post-translational modifications (PTMs) into the peptide sequence in a stepwise and regulated fashion. In spite of its many advantages one major disadvantage of SPPS is its length limitation. SPPS can only synthesize peptides restricted to ~50 amino acid residues in length [2]. This length limitation of SPPS can be attributed to the formation of intermolecular aggregates, addition/deletion of amino acids from the growing peptide chain, and a higher number of side reactions as the peptide chain increases in length [3,4]. Native Chemical Ligation (NCL), developed by the Kent laboratory [5], allows for the construction of longer peptide chains while retaining the native peptide backbone, which for many peptides is crucial for maintaining bioactivity. The basis of this chemical approach allows for the coupling of two individual fully deprotected peptide fragments in an aqueous solution to form a site-directed native peptide bond at the ligation site. NCL in conjugation with SPPS can be used to make functional proteins that are ~250 amino acids in length [6]. Peptides and proteins play critical roles within biological and physiological processes in all living organisms. The chemical synthesis of peptides has been developed for over 100 years [6]. During this period, NCL was one of the most important breakthroughs in peptide chemistry. In this review we discuss the evolution, advantages and potential applications of this technique.

## 2. History of Ligation Strategies

### 2.1. Hydrazone Ligation

One of the earliest forms of ligation [7] utilized a hydrazone linkage, an amine derivative of an imine. This bond is typically created by reacting a hydrazine moiety with an aldehyde resulting in a nucleophilic attack on the carbonyl carbon and the formation of the hydrazone bond through the loss of water. This highly chemo-selective reaction negates the need for side-chain protection groups, allowing molecules to remain small and water-soluble. However, this method is slow, typically taking 1–3 days to go to completion but can be accelerated through the use of polar, aprotic co-solvents like dimethyl sulfoxide (DMSO). This technique has been used by Cabezas *et al.*, to produce a synthetic HIV-1 vaccine [8] as well as peptide dendrimers [9] (refer to Table 1).

### 2.2. Oxime Ligation

An oxime bond can be created by reacting an oxyamine with a free carbonyl under acidic conditions. This reaction proceeds at a modest rate under acidic conditions. However, improved kinetics can be achieved either by using millimolar concentrations of each reactant or a large excess of one of the reagents. The reaction can be further accelerated by adding a nucleophilic catalyst such as aniline. This effectively changes the electrophile from a relatively unpopulated carbonyl, to the highly populated Schiff base of the aniline. This addition results in up to a 400-fold increase in the rate of oxime formation in mildly acidic aqueous conditions [10]. Oxime formation is also heavily influenced by temperature and co-solvents. Reactions conducted under high heat (37 °C) and/or using DMSO or dimethylformamide (DMF) as co-solvents saw a marked increase in oxime bond formation, as well as a substantial decrease in the number of observed side reactions in the case of the DMF [11]. Also, unlike other imines such as hydrazones, oximes are stable at physiological pH [10]. The reaction proceeds very close to completion (~90%), and the final polyoximes are easily purified and soluble in both water and phosphate buffered saline (PBS) resulting in relatively good yields [9]. Oxime ligation has been used to successfully synthesize peptide dendrimers [11] and famously by Rose to synthesize homogenous, branched polypeptides with 195 amino acid residues [9] (refer to Table 1).

### 2.3. Thiazolidine Ligation

In order to achieve the goal of overcoming the kinetic barrier of ligating two large peptides, a highly specific reaction was necessary. This was first accomplished in 1994 by Liu and Tam by reacting the alkyl aldehyde glycolaldehyde with the mercaptamine moiety of a cysteine residue that functions as a weak base. These reagents coupled with acidic conditions, which effectively excludes stronger bases, like amines and guanidino groups, from reacting. The high specificity of this reaction makes side-chain protection groups unnecessary. This arrangement enables the formation of a stable 5-membered thiazolidine ring via a dehydration reaction. Ring formation is fast and complete, typically taking ~15 min at pH 5–6 and less than 5 min at neutral or basic pH. The final step in this reaction is the *O*,*N*-acyl rearrangement. This reaction, whereby the secondary amine of the newly formed thiazolidine ring performs a nucleophilic attack on the carbonyl carbon, constitutes the rate-determining step of this process. Since the amine is a weak base, the reaction itself proceeds best under acidic conditions and is also dependent on the steric and electronic hindrances of both the carbonyl and amino functionalities.

Liu and Tam originally hypothesized that the dependence on acidic pH is likely due to the electron-withdrawing capability of the carboxamide group, which leads to a decrease in the basicity of thiazolidine amine [12]. However thiazolidine ligation is made less desirable by its reaction conditions. In order to achieve the acidity necessary for the *O*,*N*-acyl rearrangement, triflouroacetic acid (TFA) is typically used. Furthermore, a heavy metal catalyst, like Ag^+^, is also needed [13]. This methodology pioneered by Liu and Tam [12] was subsequently used by Liu *et al.*, in order to synthesize analogs of HIV-1 protease [12], as well as for the creation of peptide dendrimers [11] (refer to Table 1).

### 2.4. Thioester Ligation

This technique, developed by Schnölzer and Kent, involves a thioester fragment performing a nucleophilic attack on an acyl halide in a S_n_2-type mechanism, resulting in a ligated product that is indefinitely stable under mildly acidic conditions (pH 4.3). The reaction is rapid and goes almost to completion after 3 h, with extensive product formation detected via reverse phase-high performance liquid chromatography (RP-HPLC) after 45 min. However, due to the fact that the thioester bond is labile under basic conditions (pH > 7.5), this technique must be performed using Boc-chemistry which necessitates the use of HF (when using solution-based synthesis), or TFA (during solid phase peptide synthesis) for the deprotection step. This was used to construct HIV-1 protease from two unprotected peptide fragments [14,15]. All of the previously mentioned techniques rely on joining peptide fragments through the use of non-native bonds. However, criticism [15] of how this would affect the biological activity of the resulting peptide led to the development of Native Chemical Ligation (NCL) by the Kent lab, a technique which retains the native peptide bond between the ligated fragments, preserving the biological activity of the resulting peptide (refer to Table 1).

**Table 1:** Highlighting the advantages, disadvantages, and underlying mechanism of hydrazone, oxime, thiazolidine and thioester ligation.

Technique	Advantages	Disadvantages	Ref.
Hydrazone Ligation 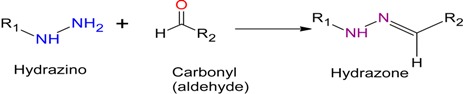	• Greater hydrolytic stability • Good water solubility • Highly chemo-selective • DMSO co-solvent accelerates reaction rate by 20× • Bond is stable at neutral pH (5–7)	• Reaction rate not affected by in pH • Rate only mildly affected by temperature (37 °C)	[7,8,9,16]
Oxime Ligation 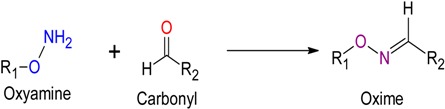	• Stable at room temp at pH 2–7 • Fragments self-assemble under relatively mild conditions • Reaction goes close to completion (~90%) • Good yield • Poly-oximes are easily purified and water soluble	• Long reaction time (16–18 h) • Basic pH leads to more side reactions	[9,10,11,16]
Thiazolidine Ligation 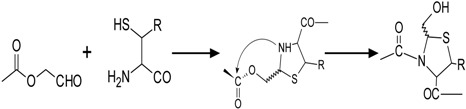	• Can create longer polypeptides (>50 residues) • Good yield • Retains K_d_ similar to native • Acidic conditions prevents side reactions of aldehydes • Ring formation is quick and complete (5–15 min) • No protection groups • Highly selective reaction	• Heavy metal catalyst needed (Ag^+^) • Highly acidic conditions (TFA) • Possible reduction in V_max_ • *O*,*N*-Acyl rearrangement is slow and dependent on stearic hindrance	[11,12,13]
Thioester Ligation 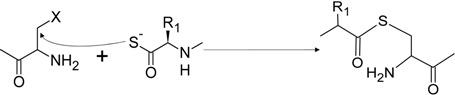	• Ligation of N and C-terminal fragments occurs rapidly via S_N_2 reaction • Ligation product was indefinitely stable at pH 4.3 (ligation conditions) • Easily purified by RP-HPLC • Ligation site can be replaced with any amino acid	• HF is necessary for Boc deprotection • Thioester bond is labile at higher pH, with hydrolysis occurring at ~7.5, leading to a half-life of about 2 h • Must account for inversion of configuration that occurs with S_N_2 reactions	[13,14,15]

## 3. Native Chemical Ligation

### 3.1. Native Chemical Ligation at Cysteine

Native chemical ligation has played a significant role in the advancement of peptide chemistry. NCL allows two peptide fragments to be joined in a covalent peptide bond at the site of ligation [5]. NCL helps overcome the shortcomings of Solid Phase Peptide Synthesis (SPPS) by allowing researchers to synthesize peptides that are greater than 50 amino acids in length [2,17]. The covalent nature of the bond at the site of ligation has been demonstrated through chemistry [5], X-ray crystallography [18] and NMR [19]. Another important feature of NCL is the absence of racemization at the site of ligation. Studies conducted by Lu *et al.*, demonstrated that the ligated product had no racemization within a limit of less than 1% D-amino acids [20]. The success of NCL can be attributed to the regioselective and chemoselective nature of the reaction. Ligation only occurs at the C-terminal thiol-ester and N-terminal cysteine thereby making the reaction highly specific and efficient. The presence of internal cysteine residues does not hinder this reaction and no additional protectional groups are required for any of the amino acids found in the protein thereby making NCL an invaluable regioselective reaction [15,21,22]. This highly selective reaction is performed in an aqueous environment at neutral pH in the presence of denaturing agents. The presence of denaturing agents like 6 M guanidine hydrochloride allows the reaction to be performed at high concentration without causing aggregation of the reactants [2,5,6].

**Scheme 1:**
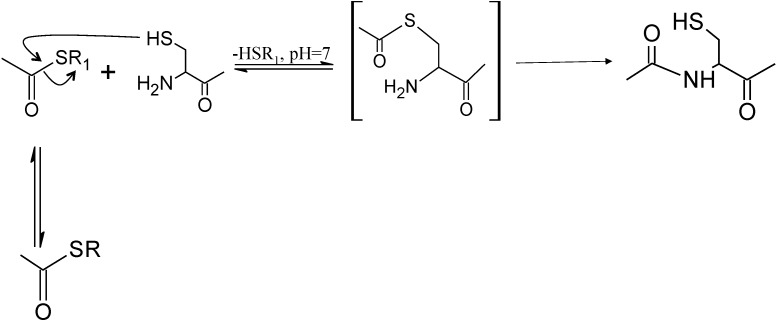
The mechanism of Native Chemical Ligation. R_1_ can be an Alkyl or an Aryl group.

An NCL reaction involves three major steps. The first step involves the thiol-thioester exchange between the thioester containing peptide fragment and externally added alkyl or aryl thiol to create a new thioester containing peptide. In the second step a transthioesterification reaction occurs between the new thioester peptide and N-terminal cysteine to produce a thioester-linked intermediate. In the third step the thioester linked intermediate undergoes a rapid intramolecular S to N acyl transfer rearrangement to form a native peptide bond at the ligation site. The first two steps in NCL involving the exogenous thio-thiol ester exchange and transthioesterification are reversible, whereas the third step, which involves the rearrangement of intermediate, is irreversible. The irreversibility of the third step and the reversible nature of the first two steps cause the freely equilibrating intermediates to be diminished over time leading to the formation of ligated product [5,23] (Scheme 1).

During the first thiol-thioester exchange the exogenous thiol could be an alkyl or an aryl thiol. However aryl thiols are preferred over alkyl thiols because aryl thiols have a lower pK_a_ and are better leaving groups. NCL reactions involving aryl thioesters can take days to go to completion, whereas reactions with alkyl thioesters happen relatively quickly [24]. Johnson *et al.* [25] examined fourteen different thiol catalysts chosen on the basis of their pK_a_ and found that aryl thiols were better catalysts than alkyl thiols. Furthermore they demonstrate that 4-mercaptophenylacetic acid (MPAA) is a non-malodorous water-soluble thiol catalyst that is highly efficient at optimizing NCL. The Kent laboratory has used MPAA in large syntheses such as HIV-1 protease [26] and RNase A [27] further validating the viability of MPAA as an efficient catalyst in NCL. Ligation rate is also dependent on the C-terminal thioester. Ligation occurs faster at less hindered amino acids and slowly at more hindered amino acids. The work of Dawson *et al.* [23,24] demonstrated that of the 20 amino acids at least 17 amino acids could be used as C-terminal thioester containing amino acids. Valine, isoleucine and proline are not suitable as C-terminal amino acids due to their slow ligation rates. NCL has undoubtedly been a boon to peptide chemistry and some of the applications are briefly discussed in this review.

### 3.2. Native Chemical Ligation at Non-Cysteine Sites: Auxiliary Mediated Ligation

**Scheme 2:**
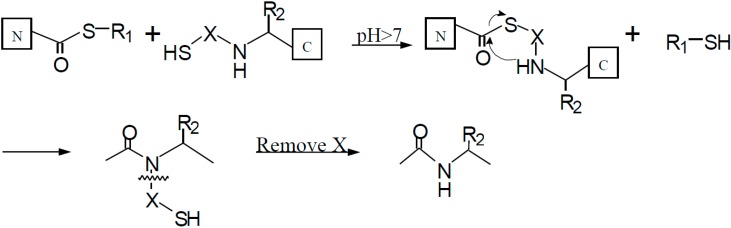
Mechanism depicting the steps of Auxiliary Mediated Ligation (AML), a technique used to join two peptide fragments resulting in large peptide molecules [36].

One of the major limitations with NCL is the requirement of cysteine for ligation. In cases where cysteine is not present or is located at a position that is not amenable for ligation, NCL loses its effectiveness [28]. Auxiliary mediated ligation (AML) was designed to circumvent this problem and this technology has been relatively successful. AML involves an auxiliary group at the N-terminus that contains a free thiol which functions to replace the thiol in cysteine. The thiol-containing auxiliary reacts with the C-terminal thioester (Scheme 2) and upon removal of the auxiliary we get the desired product. Electron-rich N^α^-benzyl type systems such as the N^α^-(1-phenyl-2-mercaptoethyl) or the 4,5-dimethoxy-2-mercaptobenzyl (Dmb)-auxiliary have been the most successful auxiliary systems [29,30,31,32,33]. Auxiliary mediated ligation has been successfully implemented in the synthesis of the electron transfer protein cytochrome b562, the 62 amino acid SH3 domain from α-spectrin, cyclic peptides, and glycopeptides [28,34,35]. A requirement of AML is the presence of glycine at one of the ligation sites due to the bulkiness of the auxiliary group.

### 3.3. Ligation at Non Cysteine Amino Acids

NCL followed by desulfurization has been used to convert a cysteine residue to an alanine residue after ligation. Since alanine is more abundant than cysteine, ligation in conjugation with desulfurization can be used to expand the amino acids amenable to the ligation process [28]. This technology was reported by Yan and Dawson who utilized this strategy to synthesize microcin J25 [37], which is a 21 amino acid cyclic peptide that has one alanine residue and no cysteine residue. A cysteine residue was utilized to perform intramolecular ligation to give a cyclic peptide. The cyclic peptide was then subjected to a desulfurization reaction to give a native sequence.

The desulfurization reaction is performed in the presence of metal reagents. Several metal reagents such as Pd/Al_2_O_3_, Pd/C, Pd/BaSO_4_ and Raney nickel have been used for the desulfurization reaction [28,37]. Cysteine residues not participating in desulfurization need to be protected during desulfurization. Kent *et al.* proposed the use of acetamidomethyl (Acm) as a protecting group for cysteine. Acm is stable to both Boc and Fmoc chemistry and can be removed using iodine [38]. One of the major problems encountered with metal-based desulfurization technology is the low yield of product, which results from peptide aggregation and adsorption on large metal surfaces. These desulfurization reactions are also prone to undesirable side reactions such as hydrogenation of tryptophan and demethylthiolation of methionine which further reduce the yield of final product [37]. Methods have been developed to eliminate the metal reagents used during desulfurization. To overcome some of the drawbacks associated with metal based desulfurization Wan and Danishefsky developed a technique for desulfarization of mercaptans by using trialkylphosphites under thermal and photochemical conditions [39].

Recent methodologies have been developed in which amino acids other than alanine can be generated through desulfurization. The technology developed by Crich *et al.* [40] and Botti *et al.* [41] uses a modified phenylalanine in which the β-carbon has mercaptan group. This modified amino acid can be used in NCL and upon desulfurization is converted to phenylalanine. The major challenge in this technique is that β-mercaptophenylalanine is not commercially available and was developed by Crich *et al.* in a five step procedure using *threo*-β-hydroxy-L-phenylalanine derivative as the starting material [40]. Modified valine has also been used in NCL. Seitz *et al.* [42] reported using β,β-dimethylcysteine as a valine alternative, whereas Yan *et al.* [37] have proposed the usage of γ-thiolated valine for NCL-desulfurization procedures. β,β-Dimethylcysteine is commercially available as peniciallamine and was used to make two 22mer spanning segments of transmembrane spanning proteins STAT-1 and Syk-kinase. In both cases radical-mediated desulfurization reactions were performed to give valine [28]. As mentioned above, various attempts have been undertaken to perform NCL at non-cysteine residues and several successful strategies have been developed to overcome the cysteine requirement for NCL. All of the above mentioned technologies have expanded the repertoire of amino acids that can be utilized in native chemical ligation.

## 4. Sequential and Convergent Ligation

**Figure 1:**
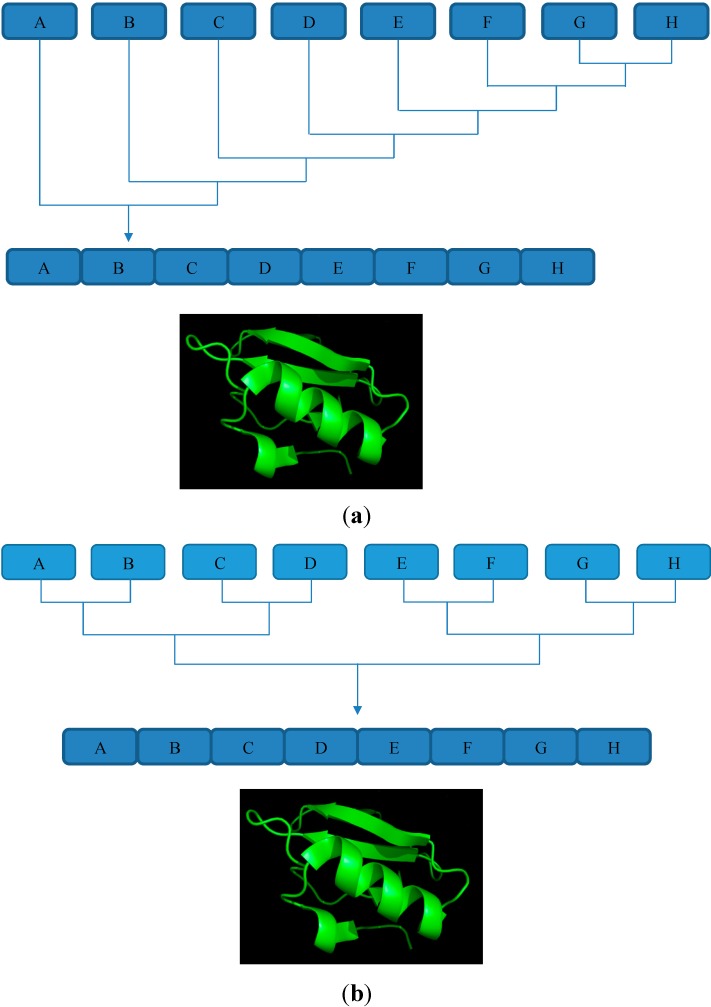
(**a**) Scheme showing sequential ligation strategy for joining peptide fragments together in order to produce a large peptide molecule [45]; (**b**) Scheme showing a strategy for ligating smaller fragments together in a convergent fashion in order to produce larger peptide molecules [45].

Native chemical ligation can be applied in the synthesis of functional proteins [15,43]. Sequential ligation and convergent ligation can be used to join multiple small fragments to give a large protein (Figure 1). Careful synthetic design and efficient planning to minimize product loss and byproduct accumulation are extremely important when undertaking such a synthetic endeavor. Small high quality fragments of up to 30 amino acids residues are more desirable than longer fragments of lower quality. Since HPLC purification and lyophilization lead to handling losses, one-pot ligation or solid phase ligation can be undertaken to avoid HPLC purification of intermediates [36,44,45].

Some of the major strategies available for sequential ligation are one-pot ligation, His_6_-tag assisted ligation and solid phase ligation. A one-pot ligation strategy was developed to eliminate purification process thereby reducing the material loss through handling. Sequential ligation of peptide fragments is undertaken in one reaction vessel to minimize handling losses. Using a 1,3-thiazolidine-4-carboxo (Thz) protecting group for the N-terminal cysteine of the peptide thioester fragment Bang *et al.* [44,46] were able to perform one pot ligation by adding methoxyamine hydrochloride and changing the pH of the reaction solution. Another strategy employed for one pot ligation is using a photolabile moiety to protect the N-terminal cysteine of peptide thioester fragment. Upon photoradiation the protecting group is removed and ligation is continued [47]. One pot sequential ligation strategy was adopted in the synthesis of crambin and ubiquitin [45]. The major drawback in one pot ligation is that byproduct and unreacted peptide fragments accumulate with each sequential reaction.

His_6_-tag ligation allows rapid purification and high recovery of target material after sequential ligation of the peptide fragment [48]. In this methodology the C-terminal peptide fragment has a His_6_ tag that binds to a Ni-nitrilotriacetic acid (NTA) agarose column. The NCL reaction can be driven to near completion by using the peptide thioester fragment in excess. An advantage of this method is that since the C-terminal fragment is attached to a resin support, a simple washing procedure is used to eliminate the unreacted thioester fragment and non-tagged fragments. Another advantage of the His_6_-tag sequential ligation technique is that it allows the intermediates in the reactions to be eluted thereby providing analytical control at various points in the ligation process. A tetratrico peptide repeated protein and a crambin protein have been successfully made through His_6_-tag ligation strategy [45,48].

Solid phase sequential ligation adopts principle from solid phase peptide synthesis. Like peptide synthesis, the growing peptide fragments are attached to a resin support (Figure 1a). Similar to peptide synthesis excess reactants and undesired byproducts can be removed through filtration. Comparable to SPPS the target polypeptide can be generated by cleaving the polypeptide from the resin support [49,50]. An acid labile safety catch linker [49] and selectively cleavable ester linker [51] are used to grow the polypeptide on a resin support. Solid phase sequential ligation strategy has been successfully employed in the synthesis of C5a (74 amino acids), MIF (115 amino acids), and human group V secretory Phospholipase A2 (118 amino acids, six disulfide bonds) [45].

Another method for joining multiple peptide fragments is through convergent ligation. Unlike sequential ligation, in convergent ligation two halves of a target sequence are ligated separately and condensed to give the target polypeptide (Figure 1b). Convergent ligation is more efficient than sequential ligation because there is a lesser chance for byproduct accumulation and purification is easier in this approach [52,53]. Initially, convergent ligation led to the formation of a non-native peptide bond at the site of ligation [54]. Researchers utilized various functional groups to generate non-native bond at ligation site. A thioester bond forming ligation was developed that used thioacid and bromoacetyl functional groups. Additionally, an oxime bond forming ligation was developed through (aminooxy) acetyl and ketone groups [45]. Baca *et al.* reported synthesizing a HIV-1 protease analogue through convergent peptide ligation. This synthetic analogue showed similar catalytic activity to the native protein but possessed a non-native bond at various ligation sites [55]. With the advent of kinetically controlled ligation researchers were able to perform convergent ligations that generated native peptide bonds at the site of ligation [52]. Kinetically controlled ligation works on the principle that peptide-α thiophenylester reacts with a Cys-peptide faster than a peptide-α thioalkylester [56]. Kent *et al.*, proposed that, in the absence of external thiophenol under competitive reaction conditions, the large difference in the reaction rates between the α-thiophenylester and α-thioalkylester would make the α-thioalkylester unreactive [45,52]. Kinetically controlled ligation has been successfully implemented in the synthesis of human lysozyme (130 amino acids) [57] and a covalent dimer of HIV-1 protease (203 amino acids) [58].

## 5. Application of Native Chemical Ligation

### 5.1. Cyclotides and Conotoxins

**Scheme 3:**
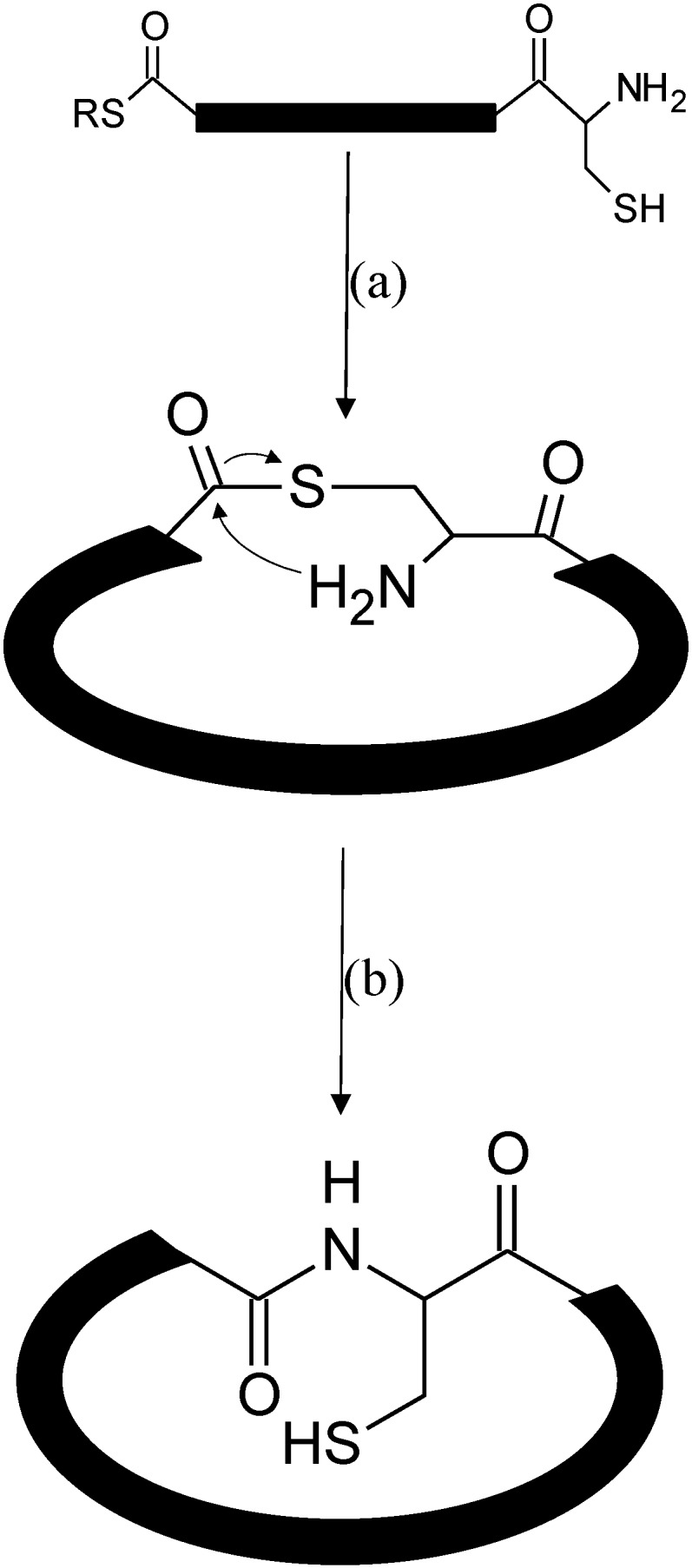
Mechanism showing the cyclization of a linear peptide. (**a**) Intramolecular nucleophilic attack results in a cyclic peptide joined by a thioester bond (**b**) S-N Acyl Shift produces the final cyclic molecule now joined by a native peptide bond.

NCL technology can be implemented to make peptides in which the amino and carboxy termini are attached in a covalent bond to form a cyclic molecule. When the thioester and free cysteine functionalities are present on the same peptide fragment, an intramolecular NCL reaction can be used to generate a cyclic molecule (Scheme 3). Recently, naturally occurring cyclic molecules have been discovered in bacteria, plants and animals [59,60]. NCL reactions can be utilized to synthesize these circular proteins to find potential new drug leads and design scaffolds that aid in drug delivery.

Cyclotides are disulfide rich compounds naturally occurring in plants. They generally have about 30 amino acids and are predominantly found in the Rubiaceae (coffee) and Violaceae (violet) families [61,62,63]. Cyclotides fall into two major subfamilies, the bracelet family that contains two-thirds of cyclotides, and the mobius family that contains the remaining one-third. There is a third subfamily called trypsin inhibitor family comprised of eight members [64,65]. Cyclotides are characterized by head to tail cyclization and contain six conserved cysteine residues arranged in a knotted topology. The cyclic cystine knot morphology provides cyclotides with exceptional stability thereby making them prime candidates for grafting studies and as templates for drug design [66,67]. These cyclic molecules have shown a vast array of activities ranging from antimicrobial, cytotoxic and anti-HIV. In nature cyclotides function as host defense agents and possess potent insecticidal activity [62,68,69,70,71].

Craik *et al.*, have used NCL to successfully conduct grafting studies on cyclotides. They combined stable cyclotide fragments with bioactive peptide epitopes to produce chimeric molecules with enhanced biopharmaceutical properties. As a proof of concept they grafted a highly polar region to a cyclotide and showed that the molecule still maintained its structural integrity [72]. Furthermore Gunasekera *et al.*, incorporated an antiangiogenic epitope (RRKRRR) referred to as the poly R sequence into kalata B1. The chimeric molecule possessed antiangiogenic activity and was more stable than the naked epitope [73]. Similarly Thongyoo *et al.*, have proposed that the active site in cyclotides loop 1 could be replaced with a sequence to inhibit a protease from the Foot and Mouth Disease virus [74]. Reengineering of cyclotides to produce chimeric molecules has shown promise in therapeutic development, and with further advancement this technology might produce future drugs.

Like cyclotides, conotoxins are natural products that are rich in cysteine residues thereby providing ample opportunities for NCL. Conotoxins are found in the venom of conesnails and act as potent antagonists of a range of neuronal receptors and ion channels [75]. ω-conotoxin MVIIA, a potent N-type calcium channel blocker extracted from *Conus magus*, was developed into ziconotide. This “conopeptide” is the first non-opioid IT treatment for the management of chronic refractory pain and is known by its brand name Prialt^®^ [76,77,78]. One of the major hurdles in developing conopeptides as therapeutics is overcoming their low oral bioavailability [65]. Peptides and proteins have low oral bioavailability due to degradation caused by digestive enzymes present in the intestinal lumen [79,80,81]. Secondly, the inability of peptides to permeate through membranes owing to size and polarity also results in low bioavailability [79,82]. Consequently any method that improves the stability and bioavailability of peptides and proteins is highly desirable. Work done by Clark *et al.*, on cyclic α-conotoxin MII [83], Clark *et al.*, on cyclic α-conotoxin Vc1.1 [84] and Lovelace *et al.*, on cyclic χ-conotoxin MrIA [85] has demonstrated that NCL can be used to cyclize conotoxins. Their work shows that when provided with a linker of right size the cyclic molecules can maintain their biological activity and are more stable than native peptides. Cyclization helps with stability by eliminating the free termini of a peptide and makes it less labile to exopeptidases. Additionally cyclization enhances stability by introducing rigidity and reducing the conformational energy of the unfolded state [86]. The added rigidity makes the peptide more resistant to proteases that cleave from the middle of the chain [87]. Conotoxins are a rich source of biological leads that might provide therapies for pain management, seizures, and Parkinson’s [84,88]. Through cyclization we can improve the therapeutic potential of conotoxins and enhance the number of potential leads in our pharmacological repertoire.

### 5.2. NCL in Overcoming Length Limitation Associated with SPPS

SPPS is a great tool for making peptides with non-natural amino acids or incorporating post-translational modification(s). However the size limitation imposed by SPPS puts constraints on its usefulness. SPPS in conjugation with NCL can be used to overcome this hurdle. Initial ligation techniques introduced a non-native bond at the site of ligation (Section 1) but with the development of NCL ligations, the introduction of native bonds became possible.

One of the first total syntheses of a protein by chemical ligation was human immunodeficiency virus-1 protease (HIV-1 PR) [14]. The 22.5 kDa HIV-1 PR protein is a homodimer of two 99 amino acid polypeptide chains. The monomer was prepared by chemical ligation of a 51-residue peptide containing a C-terminal thioacid and a 48-residue peptide having an N-terminal alkyl bromide. However, the major disadvantage of this was producing a non-native bond to link the two unprotected peptide fragments.

With the advent of NCL the problem of non-native ligation was resolved. Human interleukin 8 (IL-8), which contains 72 amino acids was the first peptide synthesized using NCL [5]. SPPS was used separately to synthesize residues 1–33 of IL-8 with C-terminal thioester and residues 35–72 of IL-8 containing N-terminal free cysteine residue. Using NCL, a native bond was introduced at the point of ligation and the 72 amino acid peptide was generated. Human type II secretory phospholipase A_2_ (sPLA_2_), an enzyme made up of 124 amino acids that is found in the α granules of platelets and at inflammation sites, was also synthesized using NCL. sPLA_2_ was prepared by ligating two fragments, the first fragment contained residues 1 to 58 and the second fragment contained residues 59 to 124 [22]. The following example describes the chemical synthesis of a multidomain anticoagulant protein, “microprotein S”, through modular assembly of synthetic peptide segments by native chemical ligation. In this case, the native peptide bond was produced between Cys-TSR (47–79)-thioester and Cys-EGF1 (80–116) to form the first segment. After that, the Gla-rich fragment (1–46)-thioester was ligated to the segment through the creation of another native peptide bond that completed the synthesis of full-length microprotein S [89]. NCL has allowed peptide chemists to synthesize functional proteins and is undoubtedly a great tool in synthetic protein chemistry (Table 2).

**Table 2:** Examples of the application of NCL to circumvent peptide size limitation. Cysteine residues used as ligation sites are in bold and larger font. γ = Gamma carboxyglutamic acid.

Toxin/Peptide	Application	Sequence	Ref.
Human interleukin 8 (IL-8)	Chemokine, chemoattractants for leukocytes	SAKELRCQCIKTYSKPFHPKFIKELRVIESGPHCANTEIIVKLSDGRELCLDPKEWVQRVVEKFLKRAENS	[5,90]
Human group II secretory phospholipase A_2_ (sPLA_2_)	Hydrolysis of the fatty acid side chain ester bond	NLVNFHRMIKLTTGKEAALSYGFYGCHCGVGGRGSPKDATDRCCVTHDCCYKRLEKRGCGTKFLSYKFSNSGSRITCAKQDSCRSQLCECDKAAATCFARNKTTYNKKYQYYSNKHCRGSTPRC	[22]
Microprotein S	Anticoagulant cofactor activity	NSLLγγTKQGNLγRγCIγγLCNKγγARγVFγNDPγTDYFYPKYLGCLRSFQTGLFTAARQSTNAYPDLRSCVNAIPDQCSPLPCNEDGYMSCKDGKASFTCTCKPGWQG EKCEFD	[89]
Barnase (Lys^49^-Cys^49^)	Microbial ribonuclease	AQVINTFDGVADYLQTYHKLPNDYITKSEAQALGWVASKGNLADVAPGCSIGGDIFSNREGKLPGKSGRTWREADINYTSGFRNSDRILYSSDWLIYKTTDHYQTFTKIR	[24]

### 5.3. Synthesis of Challenging Sequences

In addition to being a great tool to circumvent some of the length limitations associated with SPPS, NCL is also highly useful in synthesizing challenging proteins. Membrane proteins are difficult to synthesize because of their large hydrophobic peptide segments, these segments lead to difficulties with amino acid coupling, formation of aggregates, and poor peptide purity and solubility [91]. In order to avoid these problems, NCL is applied, joining multiple segments to obtain a full length and functional peptide chain. A 136-mer mechanosensitive ion channel from *Escherichia coli* (Ec-MscL) was also synthesized using NCL [92]. This chemically synthesized membrane protein had cysteine residues which were not present in the native sequence [93,94]. The introduction of cysteine residues aids in ligation but results in the presence of non-native sulfhydryl groups in the final protein. In this case, ligation of three peptide fragments through NCL resulted in a synthetic, non-native Ec-MscL protein possessing similar ion-channel function to the native protein. Another scenario where NCL has shown its usefulness is during the synthesis of snow flea antifreeze protein (sfAFP). This cysteine-rich protein has 81 amino acids in its sequence and contains Cys residues at positions 1, 13, 28, and 43 [95]. sfAFP has unusual properties such as thermal instability, therefore NCL was the desired route for synthesis. The antifreeze protein was synthesized by ligating four unprotected peptide fragments that contained three thioesters: Thz1–12-thioester; Thzl3–27-thioester; Thz28–42-thioester; Cys43–81 [96,97]. Using convergent and sequential ligation many syntheses of long and difficult proteins have been accomplished. With further refinement and advancement this technology is bound to help further advance biomedical research (Table 3).

**Table 3:** Examples of the application of NCL to synthesize challenging peptide sequences. Cysteine residues used as ligation sites are in bold and larger font.

Toxin/Peptide	Application	Sequence	Ref.
Ec-MscL	Mechanosensitive ion channel	MSIIKEFREFAMRGNVVDLAVGVIIGAAFGKIVSSLVADIIMPPLGLLIGGIDFKCFAVTLRDAQGDIPAVVMHYGVFIQNVFDFLIVAFAIFMAIKLINKLCRKKEEPAAAPAPTKEEVLLTEIRDLLKEQNNRS	[92]
IbTx V16A/D19-Cys-4-MeOBzl	Binding to BK Ca^2+^-activated K^+^ channel (K_Ca_1.1)	ZFTDVDCSVSKECWSACKX2LFGVDRGKCMGKKCRCYQ (X_2_ = Cys-4-MeOBzl)	[98]
Snow Flea Antifreeze Protein (sfAFP)	Inhibition of Ice Crystal Formation	CLGADGAHGVNGCPGTAGAAGSVGGPGCDGGHGGNGGNGNPGCAGGVGGAGGASGGTGVGGRGGKGGSGTPKGADGAPGAP	[96,97]
KcsA (Pro^2^-Ala^2^, Gln^58^-Ala^58^, Thr^61^-Ser^61^, Arg^64^-Asp^64^)	Potassium Channel	MAPMLSGLLARLVKLLLGRHGSALHWRAAGAATVLLVIVLLAGSYLAVLAERGAPGAALISYPDALWWSVETACTVGYGDLYPVTLWGRLVAVVVMVAGITSFGLVTAALATWFVGREQERRGK	[99]

### 5.4. Use in Doing Alanine Scans to Study Structure Activity Relationships

Alanine scanning is a tool used in molecular biology and proteomics to determine the contribution of specific amino acid residues towards stability, functionality, and structure [100]. Furthermore, using alanine scanning it is possible to determine whether the specific residues contribute to bioactivity and protein-protein interactions. In this strategy, alanine is used to substitute each individual amino acid in the peptide sequence to assess the value of the substitution to the overall protein [101]. Since alanine is non-bulky, chemically inert and can mimic the secondary structure of many other amino acids with its methyl functional group, alanine is the ideal model to replace other amino acids. Additionally, substitution with alanine removes all side chain atoms past the β-carbon, thus this method can be used to infer the roles of individual side chains in the final peptide. Site-directed mutagenesis is a powerful tool for researching protein structure-activity relationships and alanine-scanning mutagenesis has been particularly successful in systematically mapping the protein-binding interface [102,103]. Traditional alanine-scanning mutagenesis is a laborious endeavor because each mutant needs to be individually produced, purified and separately assessed in structural and functional assays. Binomial mutagenesis was developed as an alternative to the traditional method. This “next generation” technology was based on *in vivo* library-based mutagenesis and was considerably more attractive because a single library simultaneously provides information about many different residues. However it is still limited by the issues of heterogeneity of proteins, protein expression and protein folding [104]. In order to circumvent the need for protein purification and biophysical analysis, shotgun scanning was developed for mapping the functional epitopes of proteins. This methodology includes the concepts of conventional alanine mutagenesis and binomial mutagenesis along with phage display technology [101].

**Figure 2:**
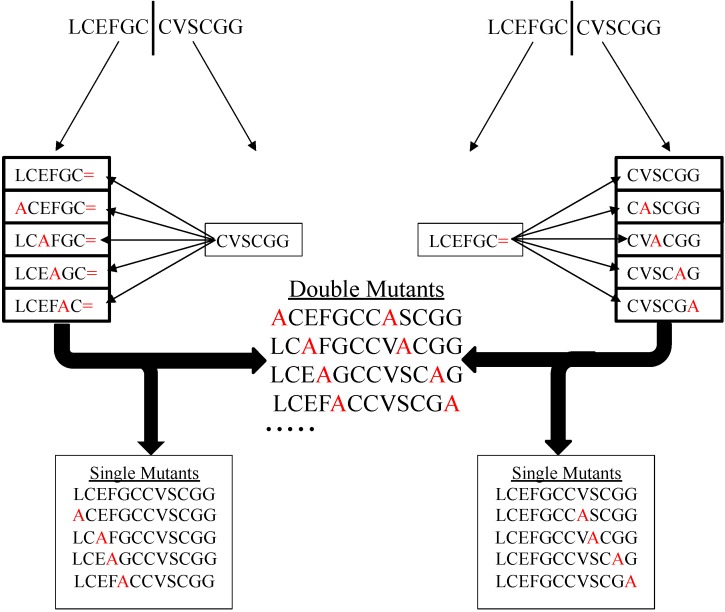
The figure demonstrates the application of Native Chemical Ligation in conducting an Alanine Scanning to study structure-activity relationships. The equal sign (=) designates a thioester functionality.

Using SPPS, researchers can synthesize different alanine substituents and one core peptide. In the next step, NCL can be used to ligate the core peptide to different fragments to generate one or two amino acid variants of the target peptide (Figure 2). NCL can be a powerful tool that can improve the efficiency of alanine scanning by: reducing the amount of required reagents, the number of syntheses, and time involved in conducting the process.

### 5.5. Conjugation with Recombinant Technology to Make Glycopeptides and Peptidomemetics

Glycoproteins have carbohydrates that are covalently attached to certain amino acid residues. A β-*N*-glycosidic linkage of *N*-linked glycoproteins connects N-acetylglucosamine to the amide residue of asparagine in Asn-Xaa-Ser/Thr motifs [105]. Alternatively an α-*O*-glycosidic linkage between the glycan and hydroxyl groups of serine, threonine or tyrosine is called *O*-linked glycosylation [106]. Mannose, xylose, fructose and *N*-acetylgalactosamine are all found to be the ligated to proteins through *O*-linked glycosylation [106]. Since many therapeutics are glycoproteins, several semi-synthetic strategies for glycoprotein preparation have been explored [107]. Moreover, recent developments in engineered cell culture employing yeast suggest that therapeutic proteins with a human-like glycosylation profile can be achieved [108].

However, human glycoproteins are heterogeneous and this level of glycosylation complexity cannot be wholly replicated in yeast recombinant systems, therefore, NCL could be an excellent platform for glycopeptide synthesis because it allows residues to be stoichiometrically and site-specifically glycosylated, while retaining native linkages. Therefore, NCL is the most widely used non-recombinant approach for the synthesis of glycoproteins. Diptericin ε, an antibacterial glycopeptide, containing 82 amino acids and two *O*-linked glycosylation sites was the first major glycopeptide synthesized using NCL. In this case SPPS was used to make two peptide fragments: the first fragment consisted of a 24-residue segment with an *N*-terminal glycopeptide thioester and the second fragment consisted of a 58-residue glycopeptide containing an *N*-terminal cysteine residue. Both fragments were covalently bonded to N-acetylgalactosamine (α-GalNAc, also known as the T_N_ antigen) at the two glycosylation sites [109].

Traditionally, the formation of thioester is based on Boc chemistry; however, the acid deprotection condition of Boc chemistry is incompatible with glycopeptide thioesters, because low pH may lead to the breakage of glycosidic linkage [105]. Therefore, a ‘‘safety-catch’’ linker was employed for the synthesis of the N-terminal glycopeptide thioester fragment to circumvent this problem [110,111]. The safety-catch linker is normally unreactive with nucleophiles in basic and acidic conditions but, after completion of the synthesis, selective sulfonamide activation renders the linker susceptible to nucleophilic attack and allows the release of the final product. Lymphotactin (Lptn), a glycoprotein with multiple *O*-glycosylation sites called ‘‘mucin type” [112], was also synthesized using NCL. Lptn is a 93-residue chemokine, which functions as a potent chemoattractant for T cells and natural killer cells [112,113]. The C-terminus of Lptn contains a mucin domain with up to eight *O*-glycosylation sites. During the synthesis of Lptn, the Lptn(1–48)-thioester fragment and the glycopeptide Lptn(49–93) fragment containing an N-terminal cysteine and eight α-GalNAc were ligated to produce a 93 amino acid glycopeptide. Synthetic Lptn was subsequently assessed for its ability to bind its cognate chemokine receptor (XCR1). The synthetic glycopeptide activated the signal transduction cascade providing an increase in intracellular calcium concentrations [105,114], thereby suggesting that the synthetic analogue was similar in function to the native glycopeptide.

The first example of using NCL to construct an *N*-linked glycopeptide, containing a complex-type glycan, was RNase B [115]. In this case, Fmoc SPPS was used for the synthesis of glycodecapeptide thioester, two linkers were used on the resin before assembling the glycopeptide. The first of these linkers was the Ellman-sulfonamide safety-catch linker, which protected the glycosidic linkage [110]. The second was a Rink amide linker, which allowed for peptides to be released during assembly in order to assess the efficiency of the synthesis by HPLC and LC-MS. The glycopeptide containing the Cys-RNase(41–68) fragment was reacted with the C-terminal thioester of the other peptide fragment to produce RNase B (Refer to Table 4).

**Table 4:** Examples of the applications of NCL to synthesize glycopeptides and glycoproteins. Cysteine residues used as ligation sites are in bold and larger font. Glycosylation sites are shown with underlining residues.

Toxin/Peptide	Application	Sequence	Ref.
Diptericin ε (Cys^25^,Glu^29^,Glu^45^)	Antibacterial glycopeptide	DEKPKLILPTPAPPNLPQLVGGGGCNRKEGFGVSVDAHQKVWTSENGRHSIGVTPGYSQHLGGPYGNSRPDYRIGAGYSYNF	[109]
Lymphotactin (Lptn)	Chemoattractant (for T- cell and natural killer cell)	VGSEVSDKRTCVSLTTQRLPVSRIKTYTITEGSLRAVIFITKRGLKVCADPQATWVRDVVRSMDRKSNTRNNMIQTKPTGTQQSTNTAVTLTG	[112]
RNase B Fragment	Cleavage of *N*-linked carbohydrates	MKSRNLTKDRCKPVNTFVHESLADVQAVCSQKNVACKNG	[115]
CCL7 (MCP-3)	Monocyte specific chemotactic protein-3	QPVGINTSTTCCYRFINKKIPKQRLESYRRTTSSHCPREAVIFKTKLDKEICADPTQKWVQDFMKHLDKKTQTPKL	[116]

## 6. Conclusions

NCL developed by the Kent lab has been an indispensible tool for peptide chemists worldwide. By overcoming the size limitation imposed by SPPS, NCL has allowed the synthesis of many proteins such as HIV-1 protease, RNase B, and Ec-MscL. Researchers have applied the NCL technology to synthesize membrane proteins and cyclic peptides. Recently there has been an increase in the number of biologics entering the therapeutic market. By applying NCL there is potential to find new protein/peptide drug leads that were previously unattainable due to their size limitation or difficulty in synthesis. NCL is one of the major achievements of recent times and has made the field of synthetic peptide chemistry more efficient and diverse.
